# E-Health: A Game Changer in Fetal and Neonatal Cardiology?

**DOI:** 10.3390/jcm12216865

**Published:** 2023-10-30

**Authors:** Paul Padovani, Yogen Singh, Robert H. Pass, Corina Maria Vasile, Lynne E. Nield, Alban-Elouen Baruteau

**Affiliations:** 1 CHU Nantes, Department of Pediatric Cardiology and Pediatric Cardiac Surgery, FHU PRECICARE, Nantes Université, 44000 Nantes, France; padovanipaul17@gmail.com; 2 CHU Nantes, INSERM, CIC FEA 1413, Nantes Université, 44000 Nantes, France; 3 Division of Neonatology, Department of Pediatrics, Loma Linda University School of Medicine, Loma Linda, CA 92354, USA; 4 Division of Neonatal and Developmental Medicine, Department of Pediatrics, Stanford University School of Medicine, Stanford, CA 94305, USA; 5 Department of Pediatric Cardiology, Mount Sinai Kravis Children’s Hospital, New York, NY 10029, USA; pediheart@gmail.com; 6 Department of Pediatric and Adult Congenital Cardiology, University Hospital of Bordeaux, 33600 Bordeaux, France; corina.vasile93@gmail.com; 7 Division of Cardiology, Labatt Family Heart Centre, The Hospital for Sick Children, University of Toronto, Toronto, ON M5S 1A1, Canada; 8 Sunnybrook Health Sciences Centre, Toronto, ON M4N 3M5, Canada; 9 CHU Nantes, CNRS, INSERM, L’Institut du Thorax, Nantes Université, 44000 Nantes, France; 10 INRAE, UMR 1280, PhAN, Nantes Université, 44000 Nantes, France

**Keywords:** congenital heart disease, e-health, telemedicine, mobile health, artificial intelligence

## Abstract

Technological advancements have greatly impacted the healthcare industry, including the integration of e-health in pediatric cardiology. The use of telemedicine, mobile health applications, and electronic health records have demonstrated a significant potential to improve patient outcomes, reduce healthcare costs, and enhance the quality of care. Telemedicine provides a useful tool for remote clinics, follow-up visits, and monitoring for infants with congenital heart disease, while mobile health applications enhance patient and parents’ education, medication compliance, and in some instances, remote monitoring of vital signs. Despite the benefits of e-health, there are potential limitations and challenges, such as issues related to availability, cost-effectiveness, data privacy and security, and the potential ethical, legal, and social implications of e-health interventions. In this review, we aim to highlight the current application and perspectives of e-health in the field of fetal and neonatal cardiology, including expert parents’ opinions.

## 1. Introduction

Congenital heart diseases (CHD) affect approximately 1% of births worldwide and remain a major cause of childhood morbidity and mortality, posing significant challenges to healthcare resources [1,2]. Children with CHD often require multiple surgical interventions and are exposed to long-term cardiovascular risk and neurodevelopmental disorders. While 90% of CHD children reach adulthood, new challenges remain to improve health-related quality of life (HRQOL), promote therapeutic education, and provide long-term support to these patients. The challenge of the last forty years has been to build up and improve neonatal cardiac surgery programs, whereas today’s goals relate to the neurodevelopment of children operated on in the first years of life, as well as the quality of life of their families [3,4,5,6,7,8].

Fetal and neonatal cardiology is a rapidly evolving field that increasingly relies on advanced technology to provide high-quality care to patients [9,10]. E-health has the potential to revolutionize the management of CHD throughout a patient’s life, from antenatal screening to transition to adulthood, by enhancing diagnosis, monitoring, and management of heart disease [9]. E-health encompasses various applications, including telemedicine, mobile health (mHealth), electronic health records (EHRs), and artificial intelligence (AI), each of which has potential benefits and limitations that need to be explored further [11]. This review aims to provide a comprehensive assessment of the current state of e-health in fetal and neonatal cardiology, discussing the various applications of e-health in different clinical scenarios, potential challenges, and ethical considerations. Throughout this review, we include the views of expert patients to provide a patient-centered perspective on the advances and potential benefits of e-health applications in the field.

## 2. Background

### 2.1. Definitions

E-health, denoting the application of information and communication technology to facilitate or enhance healthcare provision, represents a wide-reaching concept encompassing a multitude of endeavors associated with internet-based healthcare. This encompasses telemedicine, telecare, mobile health (mHealth), and electronic health records (EHRs), and increasingly incorporates artificial intelligence (AI) as well [12].

Telemedicine relates to the use of technology to provide medical care remotely. In pediatric cardiology, telemedicine has been used for remote consultations, follow-up visits, and even for remote monitoring of CHD infants [9]. Telemedicine has been particularly useful in improving access to care for patients living in rural or underserved areas [13]. Furthermore, telemedicine has been shown to reduce healthcare costs and improve patient satisfaction [14].

mHealth refers to the use of mobile devices, such as smartphones and tablets, for healthcare purposes. In pediatric cardiology, mHealth has been used for patient education, medication adherence, and even for remote monitoring of vital signs [13]. mHealth applications have been shown to improve patient outcomes and reduce hospital readmissions [15].

EHRs are digital versions of a patient’s medical records. EHRs have been shown to improve the accuracy and completeness of medical records, leading to improved patient care. EHRs also allow for easier sharing of medical information between healthcare providers, which can lead to better coordination of care and improved patient outcomes [16].

Artificial intelligence (AI) and machine learning (ML): AI pertains to addressing challenges or attaining knowledge through computational algorithms akin to human cognitive processes. ML constitutes a subset of AI dedicated to acquiring, deriving rules from, and drawing inferences and predictions from accumulated data [17].

### 2.2. The Need

With improvements in information technology, adjunctive e-health is very much needed in the modern clinical medicine practice, especially in subspecialties like pediatric cardiology. In many parts of the world, pediatric cardiology services are centralized to deliver high-quality patient care by experienced physicians. This makes the patients (children) and their parents travel long distances and miss school and work days, and puts some pressure on limited travel resources [18]. E-health with telemonitoring and e-health consultations can help in minimizing these long-distance travels. Information technology is already there or evolving very fast to support the safe delivery of e-health in pediatric cardiology. Already, distance cardiac monitoring, including ambulatory ECG and recognition of arrhythmia, is currently being used in most parts of the world. However, to move forward with e-health, it needs careful planning of the clinical services and resources needed to deliver such services safely. The provision of e-health may not be seen ‘physically in the clinical or hospital settings’, but it needs significant resources to develop high-quality services, and appropriate staffing to support distance monitoring; provide timely recognition of concerns; reassure patients and their parents; and when needed, to timely appropriate actions. 

### 2.3. The Past: Proof of the Concept

In tracing the history of telehealth development in fetal medicine and pediatric cardiology, we find a compelling narrative of progress and potential. The journey began with establishing telehealth services primarily focused on ambulatory ECGs (electrocardiograms) and cardiac monitoring to recognize arrhythmias. These services have become well established, providing invaluable support to healthcare providers and patients.

Fetal tele-echocardiography has demonstrated its ability to enhance the early detection of critical congenital heart disease during prenatal care. The study conducted by Sharma et al. [19] reports that effective screening for fetal heart conditions is achievable through telemedicine-assisted fetal cardiac screening and counseling, even in cases where direct personal contact with a specialist is lacking. This finding underscores the community’s acceptance and feasibility of such telehealth practices for 20 years.

## 3. Current Applications in Fetal Cardiology

### 3.1. Education, Teaching, and Training

**Figure 1:**
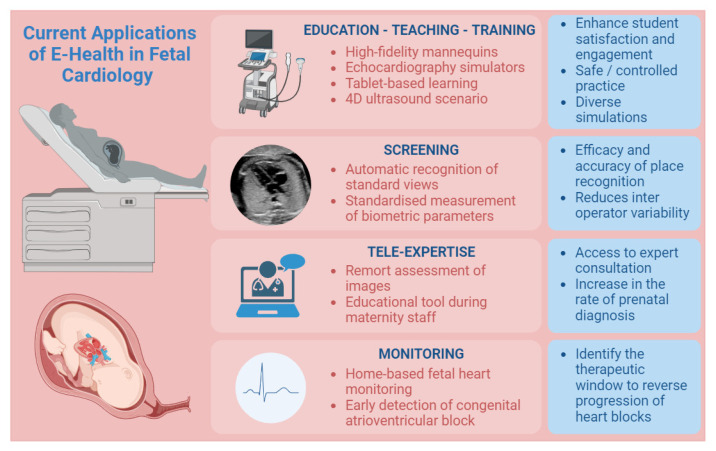
Overview for telehealth applications in the field of fetal cardiology. Legend: this figure demonstrates an extensive outlook for applications in the field of fetal cardiology and expected benefits for patients, family, or practitioners.

Echocardiography remains the foremost noninvasive modality for antenatal screening of CHD. However, antenatal screening encompasses multiple tiers, ranging from primary obstetrician consultations to tertiary fetal healthcare facilities. The scarcity of experts in CHD, coupled with the extensive and demanding training required for antenatal screening, faces significant challenges. To address these obstacles, simulation-based approaches have emerged as a valuable tool for medical education, allowing trainees to engage with virtual patients within a less stressful and more conducive environment for knowledge acquisition (Figure 1) [20]. Various contemporary learning methods, encompassing diverse levels of complexity, have been employed, including high-fidelity mannequins, echocardiography simulators, and cost-effective alternatives such as tablet-based learning of clinical cases [21].

The use of training simulators has expanded due to factors such as reduced working hours, time constraints for training, evolving ethical perspectives, societal expectations, and economic pressures. Moreover, these devices offer the added advantage of enhancing student satisfaction and engagement [22,23]. Echocardiography simulators facilitate safe and controlled practice of essential echocardiographic techniques, encompassing probe manipulation, image acquisition, and interpretation. They also enable the simulation of diverse interventions, including needle aspirations, catheterizations, and even surgical procedures, fostering hands-on experience and procedural proficiency development [24].

The advent of a novel ultrasound simulation application introduces enhanced capabilities for incorporating a wide array of ultrasound recordings from various clinical scenarios. This application enables users to load both normal and abnormal cases, providing a realistic demonstration of anatomical anomalies and physiological structures. Comprehensive scanning of integrated cases, including exceptionally rare instances, allows users to proactively familiarize themselves with unusual scenarios. By leveraging the application’s realistic depiction of malformations and physiological anatomy, students can refine their recognition of fundamental physiological structures specific to pediatric cardiology. This immersive learning experience effectively bridges the gap between theoretical knowledge and practical application, equipping learners with the necessary skills to accurately identify and analyze CHD.

Furthermore, the application provides residents with an opportunity to deepen their understanding of characteristic features pertaining to various pathologies encountered in pediatric cardiology, thereby facilitating more informed clinical decision making. Additionally, its compatibility with powerful processors in present and future smartphone generations opens avenues for the integration of 4D ultrasound recordings, enabling the visualization of dynamic elements like beating hearts and blood vessels. This potential enhancement further augments the realism and fidelity of the simulation, maximizing its educational value [25].

### 3.2. Screening

Fetal ultrasound encounters notable challenges within the clinical pipeline, including factors such as heightened fetal mobility, increased abdominal wall thickness in pregnant individuals, and inter-observer discrepancies [26]. To address these challenges, the integration of AI into fetal cardiac ultrasound has garnered attention, focusing on automatic recognition of standard views, standardized measurement of biometric parameters, and intelligent disease diagnosis. Research indicates that the combination of AI and prenatal ultrasound yields substantial enhancements in the efficacy and accuracy of plane recognition reduces inter-operator variability and ensures consistency and repeatability in plane acquisition [27,28,29]. In a study by Arnaout et al., an integrated neural network model was trained using 1326 2D ultrasound grayscale images to discern normal hearts from complex CHD across the recommended five standard cardiac views [30]. Yeo et al. [31] developed a fetal intelligent navigation echocardiogram (FINE) in conjunction with Virtual Intelligent Sonographer Assistance (VIS-Assistance^®^), enabling clinicians to identify seven anatomical landmarks through prompted guidance. The software automatically generated nine standard fetal echocardiographic views within seconds and intelligently identified surrounding anatomical structures using Vis-Assistance [31,32,33,34]. The implementation of AI has demonstrated significant clinical potential in the diagnosis of CHD, reducing training periods, and mitigating subjective variability among clinicians [35]. In this context, artificial intelligence (AI) has been newly incorporated into the equipment used in the United States. One application of this AI software, known as Heart Assist™ (Samsung Medison Co., Ltd. in Seoul, Republic of Korea), exhibits the capability to identify fetal cardiac structures and conduct automated assessments of anatomical and functional parameters, consequently diminishing scan duration and minimizing disparities in measurements between different observers [36,37,38]. 

### 3.3. Tele-Expertise

Tele-echocardiography entails the remote assessment of images obtained from geographically distinct locations through an established communication platform [39,40]. This technology facilitates access to expert consultation and interpretation from cardiologists for healthcare providers in remote or underserved regions, aiming to mitigate neonatal mortality and surgical morbidity associated with critical cardiac conditions [41,42]. The utility of tele-echocardiography is evident in countries with extensive territories like the United States or Canada, as exemplified by Meiman et al. [43], who successfully implemented four fetal tele-echocardiography sites in Kentucky regional hospitals, resulting in a fourfold increase in the rate of prenatal diagnosis [43,44]. Furthermore, its value extends to settings where limited resources contribute to delayed diagnosis and acute presentations among children with CHD. Notably, cardiovascular conditions tend to receive relatively lower priority among primary health concerns in such contexts. Muhame et al. [45] demonstrated enhanced healthcare accessibility in resource-constrained areas by engaging remotely based pediatric cardiologists to review limited imaging from sub-Saharan Africa, the Middle East, and South Asia. Telediagnosis has also experienced growth, providing a convenient means for maternity hospital staff to obtain specialist support in the diagnostic process. Moreover, it serves as an educational tool, enabling improving skills among maternity staff through interactive discussions with specialists.

### 3.4. Patient Care 

Congenital atrioventricular block (CAVB) is a rare condition occurring in approximately 1 in 20,000 live births, but it carries significant risks such as intrauterine fetal death, neonatal morbidity, mortality, and long-term complications [46,47]. Fetal arrhythmias can lead to fetal hydrops or cardiac dysfunction, necessitating specialized monitoring and treatment. While continuous daily monitoring of fetal heart rate (FHR) at a medical facility could potentially enhance the detection of emergent CAVB, its feasibility is limited [48]. Detecting and treating emergent CAVB have been reported to restore sinus rhythm [49,50]. Notably, Cuneo et al. [51] demonstrated the feasibility, low false positive rate, and empowerment of mothers in utilizing home-based fetal heart monitoring during pregnancy. In cases of CAVB, the critical period for second-degree AVB to progress to irreversible third-degree AVB coincides with the optimal timeframe for effective intervention, which is less than 24 h [52,53,54]. By implementing twice-daily home monitoring, mothers can detect second-degree AVB and identify the therapeutic window for successful intervention to reverse the progression towards third-degree AVB [55]. 

## 4. Current Applications in Neonatal Cardiology

### 4.1. Education, Teaching and Training

**Figure 2:**
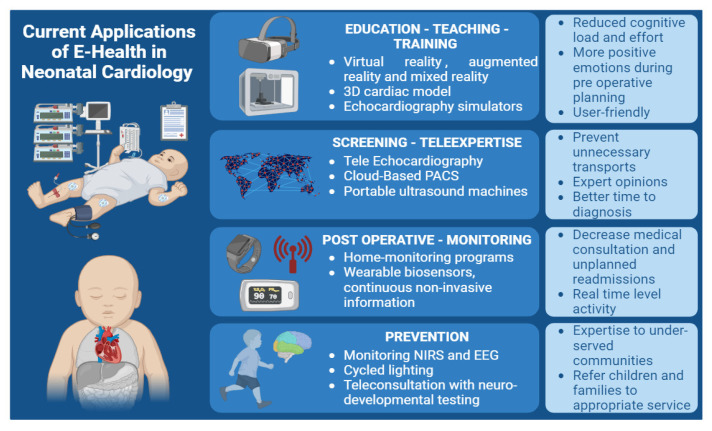
Overview of telehealth applications in the field of neonatal cardiology. Legend: this figure shows an extensive outlook for applications in the field of neonatal cardiology and expected benefits for patients, family, or practitioners.

Cardiovascular anatomy in CHD is intricately complex, and a dynamic ever-changing cardiovascular physiology during the neonatal period adds another layer of complexity. Achieving and maintaining high standards of cardiovascular care for diagnostic and therapeutic approaches necessitate extensive education and training. With technical advancements and ethical considerations, simulation has emerged as an indispensable pillar of medical education. The well-known adage “never the first time on the patient” underscores the critical role of simulation-based teaching in medicine [56]. Virtual reality (VR), augmented reality (AR), and mixed reality (MR) have emerged as innovative tools in the realm of cardiovascular care, offering valuable benefits for both healthcare providers and patients (Figure 2) [57]. VR technology completely immerses users in a virtual three-dimensional (3D) environment using a head-mounted display that covers the entire field of view. On the other hand, AR combines digital content with the physical learning environment, providing a unique advantage [58]. The utilization of VR, AR, and MR has significant potential in facilitating effective knowledge acquisition [59]. Triberti et al. demonstrated that MR technology yielded reduced cognitive load and effort, shorter response times, and elicited more positive emotions during preoperative planning [60].

The utilization of 3D cardiac models has demonstrated its significance in elucidating the intricacies of complex congenital heart anatomy preoperatively and in enhancing parents’ understanding of the CHD [61]. These models, fabricated through 3D printing techniques, offer advantages such as straightforward manufacturability, cost-effectiveness, and ease of preservation. Consequently, they hold the potential to significantly enhance the comprehension of complex structural manifestations in CHD. The implementation of this educational tool proves versatile, catering to various healthcare professionals. Specifically, it finds utility in training resident pediatricians, junior surgeons, nurses, and parents, who perceived 3D models as more readily comprehensible and user-friendly compared to conventional medical images, such as echocardiography [62]. Notably, these 3D models play a significant role in fostering parent–cardiologist interaction and bolstering engagement, thereby facilitating effective communication. Furthermore, the potential impact of this tool extends to fostering positive psychological adjustment in both parents and patients coping with the challenges of living with CHD [63,64].

Simulators are increasingly used for training in echocardiography, and there is a growing body of literature for guidelines and expert consensus recommendations in the subject [65,66]. Noori et al. [67] have demonstrated that a simulator enhances echocardiography training and decreases skill acquisition time in novice learners. In adults with congenital heart disease units, there is a growing interest in three-dimensional printing, holograms, computational modeling, and artificial intelligence in the hands of healthcare providers to better manage their comorbid patients [68,69,70,71]. As far as we know, there is currently no simulation-based learning specifically designed for percutaneous procedures in pediatric cardiology, such as the balloon atrial septostomy, despite the clear need for such a training approach.

### 4.2. Screening

Facilitating neonatal healthcare providers in employing point-of-care cardiac ultrasound for guiding hemodynamic management, while simultaneously minimizing the risk of overlooking structural anomalies, poses a significant challenge [72]. In this context, advancements in telemedicine can serve as a valuable support system to aid NICU practitioners in acquiring new skills. Moreover, telemedicine offers a means of accessing specialized advice from tertiary centers, particularly in complex cases of heart diseases and those with hemodynamic instability, before contemplating potential transfers. One available approach to enhance real-time interaction and guidance during echocardiography is through the implementation of ‘tele-echocardiography’, where a sonographer captures images at the patient’s bedside while a cardiologist remotely reviews the images in real-time, providing instructions for image acquisition and optimization [73]. Additionally, utilizing cloud-based picture archiving and communication system (PACS) platforms enables seamless transmission and viewing of stored echocardiograms on any computer, from any location [74]. Neonatal telemedicine has proven to be accurate and cost-effective, and has effectively prevented unnecessary transports in up to 75% of cases [74,75]. In remote and less densely populated regions, a substantial number of neonates encounter challenges in promptly accessing local pediatric sonographers and pediatric cardiologists skilled in echocardiographic interpretation. This situation can lead to compromised echocardiogram quality, delays in initiating medical interventions, unnecessary patient transfers, and escalated healthcare costs [76,77]. Various studies have explored the effectiveness of transmitting echocardiographic images remotely through telemedicine links for diagnosing and managing suspected CHD in neonates [78,79,80,81,82]. In the context of acutely ill infants, the availability of timely expert opinions that influence patient management can yield significant clinical advantages. A pivotal investigation conducted by Webb in 2013 demonstrated that telemedicine led to notable reductions in transport to tertiary care centers, time to diagnosis, average length of hospital stays, and durations of intensive care unit confinement [83]. This assertion has been reaffirmed through subsequent years of research [84]. This approach helps provide early targeted intervention and transfer patients to appropriate tertiary care centers such as cardiac surgical or extracorporeal membrane oxygenation (ECMO) centers.

The integration of portable ultrasound machines, traditionally reserved for emergency medicine and adult cardiology, is gradually becoming more prevalent in neonatal medicine [85]. Some manufacturers have developed probes tailored specifically for neonatal care; however, implementing such novel devices necessitates rigorous evaluation [86,87,88]. 

As the practice of echocardiography undergoes substantial transformations in neonatology, there are also significant developments in the acquisition of imaging techniques. Deformation-based assessments, such as speckle tracking and tissue Doppler, offer additional quantitative metrics for evaluating myocardial systolic and diastolic function [88,89]. Their capability to provide relatively load-independent measures of these cardiac functions stands to augment bedside hemodynamic assessment through ultrasound [90]. More recently, emerging technologies, such as three-dimensional echocardiography [91] and blood flow pattern visualization [92,93,94], offer substantial hemodynamic insights for the future. While some of these techniques, such as speckle tracking, need special software necessitating offline processing, AI and technical advancement may help in utilizing them at bedside in the near future.

### 4.3. Care Management

Recent advancements in cardiothoracic surgical techniques and medical therapies have significantly improved the management of CHD in neonates. As a result, procedural morbidity and mortality have notably decreased and outcomes have improved. Nevertheless, the initial six-month period following neonatal cardiac surgery remains a high-risk phase for both infants undergoing surgery and challenging for their parents.

During this critical period, cardiovascular morbidity and mortality are substantially prevalent. Neonates risk experiencing major cardiovascular events, such as cyanosis, rhythm disturbances, thromboembolic complications, and surgical site infections, leading to emergency room visits, rehospitalizations, and unplanned readmissions [94]. This phase is marked by heightened parental anxiety, akin to post-traumatic stress disorder, contributing to increased medical consultations, unplanned readmissions, and diminished quality of life for the parents and siblings [94]. Specific heart conditions, including single ventricle palliated heart disease, entail multistage surgical and interventional procedures, involving at least two neonatal cardiac surgeries. Therefore, the postoperative risk period is prolonged. For instance, following the Norwood procedure, ambulatory mortality during the interstage period in single ventricular cases ranges from 12 to 20% [95,96]. To address the higher mortality rate observed during the interstage period, some centers have opted not to discharge neonates after the first stage of surgery. Instead, they maintain continuous surveillance in the hospital for 4 to 5 months until the second stage of surgery, thus reducing mortality during this crucial period [97]. In Anglo-Saxon countries, an alternative approach has been developed as a home monitoring program specifically designed for these vulnerable infants [98]. This program involves close and standardized home monitoring, including daily weight and arterial oxygen saturation measurements by the parents, weekly visits or telephone calls from a pediatric nurse, and monthly consultations with a pediatric cardiologist. These outpatient follow-up programs have effectively reduced mortality by enabling early detection and management of postoperative cardiovascular complications following neonatal cardiac surgery. Numerous teams have subsequently developed interstage monitoring programs, yielding beneficial effects on interstage mortality and postprocedural weight gain [99,100,101,102,103].

Traditional clinical status and physiological assessment measures have typically been conducted within healthcare settings, offering episodic and short-term data acquired at rest. However, the emergence of wearable biosensors presents a promising opportunity to obtain continuous and noninvasive physiological information from patients with CHD in real-world scenarios. These wearable devices allow for data collection over extended periods and across varying activity levels. The advent of smart technologies has paved the way for innovative diagnostic approaches in the field of pediatric cardiology, with a notable increase in the use of wrist-worn wearables such as the Apple Watch (Apple Inc., Cupertino, CA, USA) and Fitbit (Fitbit Inc., San Francisco, CA, USA).

Among these devices, the Apple Watch has been the subject of several studies investigating its potential contributions. Kobel et al. conducted a study demonstrating the comparable quality of the Apple Watch (series 4 and above) derived iECG to a lead I in 12-lead ECG in children of all age groups, whether they had a normal heart or CHD [102]. Similarly, Paech et al. demonstrated that the Apple Watch iECG can record an ECG similar to that obtained from the standard 12-lead ECG, even in preterm neonates. [103]. Moreover, the latest version of the Apple Watch (Series 6 and above) includes a pulse oximeter feature to estimate blood oxygen saturation on demand [104]. This involves placing the watch on the skin, typically the wrist, and remaining still for 15 s while the measurement is taken. Littell et al. evaluated the use of the Apple Watch 6 pulse oximetry and ECG in a diverse pediatric cardiology population, with a significant proportion of patients having a known cardiac history. The study demonstrated good correlation and agreement between the Apple Watch 6 and a standard hospital pulse oximetry machine in this context. Other solutions, like those offered by AliveCor company (AliveCor Inc., Mountain View, CA, USA) with the KardiaMobile ECG and the KardiaPro telemedicine program showed the feasibility of a remote patient monitoring program in the Netherlands for managing arrhythmia, heart failure (weight) and blood pressure in symptomatic ACHDs [105]. Studies are needed to extend these findings to the neonatal population further.

## 5. Prevention

Advances in the medical and surgical management of infants with CHD have created an urgent need to address the long-term sequelae of CHD, with neurodevelopmental impairment now recognized as the most common morbidity experienced by infants who undergo cardiac surgery [106]. More than half of those with complex CHD will demonstrate some form of neurodevelopmental, neurocognitive, and psychosocial dysfunction requiring specialized care and impacting long-term quality of life. Preventing brain injury and treating long-term neurologic sequelae in this high-risk clinical population is imperative for improving neurodevelopmental and psychosocial outcomes [107,108]. To minimize perioperative neurologic injury risk, many centers have adopted the use of continuous, noninvasive monitors that provide surrogate markers of oxygen delivery, such as near-infrared spectroscopy (NIRS) and continuous video-EEG; this may also include monitoring for seizure activity [109]. However, despite data showing an association of reduced rSO2 and seizures with poorer neurodevelopmental outcomes, there is a lack of evidence that the incorporation of these monitoring strategies improves neurodevelopmental outcomes. Although perioperative management is critical for mitigating neurologic risk, operative and postoperative factors explain 5% of neurodevelopmental outcome variance, whereas preoperative and patient-specific factors account for 25% [110]. Recently, individualized, family-centered developmental care has been identified as a promising neuroprotective model for the cardiac intensive care environment [111]. Simple and easy modifications include cycled lighting to maintain circadian rhythms and music exposure to minimize noxious stimulation. Growing evidence suggests that long-term behavioral, social, and emotional difficulties in children with CHD may be partially attributable to parental mental health beginning prenatally [112,113,114]. Parental mental health interventions are necessary to support CHD families and optimize neurodevelopment, and new technology could be used to target disease knowledge and self-management behaviors. The Cardiac Neurodevelopmental Outcome Collaborative is a non-profit organization founded in 2016 (www.cardiacneuro.org, accessed on 27 July 2023) to “optimize neurodevelopmental and psychosocial outcomes for all individuals with CHD across the lifespan through clinical, quality improvement, and research initiatives” [115]. This American team showed the possibility to carry out standardized, performance-based neurodevelopmental testing with children and adolescents via telehealth, but none had completed comparable testing with infants and toddlers [115]. 

Based on information gathered during the intake, an assessment can be conducted via telehealth or in person; or with components of both. For patients with prior neurodevelopmental diagnosis, previous assessments, or established intervention services, a targeted telehealth test battery with a comprehensive caregiver/patient interview and record review may be sufficient to update recommendations for clinical management. For patients requiring in-person testing, an initial telehealth screening may inform the testing needed for the in-person session, reducing the length, and resources required for this visit. Telehealth may expand the footprint and breadth of cardiac neurodevelopmental care by bringing expertise to underserved, under-resourced, and remote communities and efficiently directing children and families to the most appropriate services. This also can help in reducing the cost of healthcare and improve efficiency. 

## 6. Perspectives

### 6.1. Current Limitations to Be Tackled

One of the main limitations of integrating e-health technologies into fetal and neonatal cardiology is the inability to perform a complete physical examination remotely. Physical examination findings, such as auscultation of heart sounds or palpation of the abdomen, provide critical diagnostic information that cannot be adequately assessed through virtual consultations.

Effective communication is essential for accurate diagnosis and treatment in fetal and neonatal cardiology. However, integrating e-health technologies may introduce challenges in communication, particularly in complex medical discussions or sensitive conversations with parents or caregivers [116].

Implementing e-health technologies in fetal and neonatal cardiology raises concerns regarding remuneration for physician consultations. The reimbursement models and policies for virtual consultations may vary across healthcare systems and regions, potentially impacting the feasibility and sustainability of e-health integration. Addressing these financial considerations is crucial to ensure equitable access to care and encourage widespread adoption of e-health technologies. Funding and billing for e-health need careful consideration at all levels, from providers to national policy makers.

E-health initiatives are built on reliable internet access and technological devices such as smartphones or computers. However, in resource-limited settings or families without access to broadband internet, cell phones, or computers, integrating e-health technologies may be challenging or even inaccessible. This limitation highlights the importance of addressing the digital divide to ensure equitable access to e-health solutions in fetal and neonatal cardiology.

The integration of e-health technologies requires the collection and transfer of confidential patient data. Ensuring privacy and data security is crucial to maintain patient confidentiality and comply with relevant regulations and ethical standards [117].

The potential risks of data leakage, unauthorized access, or misuse of patient information pose challenges that need to be carefully addressed when implementing e-health solutions in fetal and neonatal cardiology (Table 1).

### 6.2. A 5-Year Vision 

In the future, wearable biosensors could potentially replace invasive hemodynamic measurements, as seen in managing adult heart failure. For instance, the CardioMEMS study utilized an implanted pressure sensor to monitor pulmonary artery pressure, significantly reducing heart-failure-related hospitalizations (37% reduction in heart-failure-related hospitalization in the CardioMEMS group). Real-time data from such implantable devices can be crucial for the early detection of hemodynamic changes and provide valuable longitudinal data to understand the cardiovascular response to interventions, medications, and physiological alterations [118]. However, currently, there is a lack of evidence in toddlers and children. 

Although traditional invasive monitoring provides valuable information in neonates, it would be extremely helpful to have contactless and wireless, particularly in extremely low-gestational-age infants with fragile skin and vessels and limited surface area to apply many monitors [119,120]. Recently, some studies have demonstrated that the continuous monitoring of vital signs and hemodynamic parameters without a contact-based method, using video camera imaging and photoplethysmography (PPG) techniques, is feasible in preterm infants [121,122]. This can be achieved by positioning a camera approximately 1 m away from the infants, enabling heart rate monitoring through plexiglass barriers or in open incubators. Aarts et al. conducted a research investigation in NICUs in California and the Netherlands to explore the feasibility and challenges of these new techniques. They included 19 infants who underwent noncontact PPG monitoring. The noncontact PPG method provided a reliable heart rate measurement for over 90% of the monitoring duration [123]. 

The future of vital sign monitoring in the hospital is wireless and telemonitoring using advanced technology, but sustainability becomes a critical concern when considering young children returning home. To address this, the concept of intelligent textile medical devices has emerged, employing sensors and intelligent fabrics to monitor bodily processes and communicate data wirelessly with information systems. By adopting this approach, continuous medical monitoring could provide a “cozy” or “comfortable” nature, transforming the medicalization into a more amiable and approachable form in the home environment. It is worth noting that smart textiles differ from wearable technologies like Fitbit or the Apple Watch, as their primary aim is to seamlessly integrate electronic components, such as sensors and RFID chips, directly into the fabric of clothing, making them an integral part of the attire rather than additional accessories. While parents may express enthusiasm for such devices within hospital settings, they might resist extending monitoring into the home environment, fearing that it could heighten parental anxiety. In addition to assuring patient safety and reliability of wireless monitoring, striking a balance between technological innovation and accommodating parental concerns will be crucial in determining the successful implementation of intelligent textile medical devices for continuous monitoring beyond the hospital environment [124].

In conclusion, wearable biosensors hold great promise for the continuous and noninvasive monitoring of patients with CHDs and other cardiovascular conditions. However, further research is needed to establish their reliability and patient safety in children and develop appropriate guidelines and standards for their use in different patient populations, particularly in neonates. Careful interpretation and utilization of the data from these devices are essential to ensure their safe and effective integration into clinical practice [125].

## 7. Precision Medicine

These new technologies can provide a robust teaching and training tool. Still, in the near future, they will also be at the heart of surgical preplanning and percutaneous interventions in children. Personalized care has been associated with improved patient outcomes, particularly in complex CHDs. This is particularly evident in most neonatal cardiac catheterization procedures, which are executed to address targeted lesions or issues, thus underscoring the significance of tailored interventions in optimizing CHD management. Indeed, invasive diagnostic procedures are less frequently employed due to the highly accurate anatomical and functional information provided by noninvasive options such as echocardiography, magnetic resonance imaging (MRI), and computed tomography (CT). Consequently, pediatric and adult cardiologists specializing in invasive catheterization must be able to interpret findings from various imaging modalities and comprehensively understand their limitations to ensure effective procedure planning and execution [126]. Both VR and AR have been successfully utilized in simulating critical steps of structural heart interventions. Initially developed for adult structural cardiology procedures such as transcatheter aortic valve replacement, valvular heart disease management, and closure of the foramen ovale [127], these innovative technologies are gradually being introduced in the field of congenital cardiology. Software applications like Mimics (Materialize, Leuven, Belgium) enable radiologists and medical engineers to employ CT scans to generate models of cardiovascular structures and malformations, which can then be imported into VR or AR holographic systems. Advancements in three-dimensional (3D) technology within the realm of CHD imaging have facilitated new approaches to the imaging and treatment of various CHD such as sinus venosus atrial septal defects [128,129,130,131,132]. However, evidence supporting its suitability in children of lower body weight is still emerging. Qiu et al. demonstrated the application of this technology in children, with a mean age of 4.5 years, with pulmonary atresia, ventricular septal defect, and major aortopulmonary collateral arteries [133]. During the procedure, the virtual heart can be superimposed on the real heart using a mixed reality helmet, aiding in determining the location of the major aortopulmonary collateral arteries (MAPCAs) in the descending aorta. In the 3D group, compared to the conventional group, the cardiac bypass time (CPB) was shorter (93.2 ± 63.8 vs. 145.1 ± 68.4 min, *p* = 0.099), and the median pre-CPB time per MAPCAs was significantly reduced [25.7 (14.0, 46.3) vs. 65 (41.3, 75.0) min, *p* = 0.031]. Unfortunately, neonatal application of this type of technology is still limited, and so far, research projects are currently ongoing but scarcely published in the literature. 

## 8. Parental Perspectives

Parent’s Perspective: viewpoint from “Petit Coeur de Beurre” (Petit Coeur de Beurre is a French national non-profit organization dedicated to improving care for children and families born with congenital heart diseases). This part has been written by parents from the association. 

As an association representing parents of children with CHD, we’ve explored the impact of e-health in fetal and neonatal cardiology. Teleconsultations and digital health technologies have stirred mixed feelings among our community.

Teleconsultations offer convenience, reducing the need for stressful medical facility visits and associated risks. Consulting healthcare professionals from home reassures parents during critical times. However, we acknowledge concerns about virtual consultations potentially hampering doctor-patient communication. In-person visits provide non-verbal cues crucial for understanding a child’s condition and its family implications.

Data security is a paramount concern. While data protection laws are appreciated, safeguarding sensitive medical information during online consultations is essential for trust.

Managing medical devices at home via e-health excites and concerns us. Empowering parents requires adequate training and support. Despite concerns, we recognize e-health’s potential benefits: improved communication with healthcare providers, easier resource access, and greater care autonomy.

Patient portals and mobile apps empower parents to manage health and access medical info. They enhance education, healthcare communication, and child health support. These tools provide medical information access, aiding informed decisions, and enable communication, prescription requests, and appointment scheduling, boosting engagement and safety. Moreover, they connect parents, offer educational materials, and guidance from healthcare providers. These technologies enhance patient satisfaction, engagement, and safety by enabling tracking of child health and medication and sending reminders.

Further research is needed to optimize fetal and neonatal care patient portals and mobile apps. As we embrace e-health, we urge consideration of our experiences. Balancing benefits, risks, and ethical e-health use in fetal and neonatal cardiology is vital for our children and families.

## 9. Conclusions

While e-health technologies’ integration in fetal and neonatal cardiology presents exciting opportunities, it is essential to acknowledge and address the limitations associated with this approach. The restrictions, including the lack of physical examination, challenges in communication, remuneration concerns, limited access to technology, and privacy considerations, should be carefully considered when implementing e-health initiatives in this specialized field. By addressing these limitations and finding innovative solutions, we can maximize the benefits of e-health while ensuring the highest standard of care for fetal and neonatal cardiology patients.

## Figures and Tables

**Table 1:** This table summarizes the applications of e-health in fetal medicine, along with the cons and potential solutions for the challenges.

Applications	Pros and Benefits	Cons and Challenges	Potential Solutions
Education Teaching Training	Simulation-based teaching enhances knowledge acquisition.	High cost and resource requirements. Lack of simulation for pediatric cardiology procedures.	Collaboration with medical schools and institutions for funding. Develop simulation-based training programs for pediatric cardiology echography and procedures.
	VR, AR, MR offer immersive educational tools.		
	3D cardiac models facilitate understanding.		
	Simulators improve echocardiography training.		
Screening	Telemedicine aids in remote echocardiography.	Limited access in remote regions Compromised echocardiogram quality in certain areas.	Expand telemedicine infrastructure. Develop robust AI for CHD.
	Efficient access to specialized advice.		
	Reduced unnecessary transport and healthcare costs.		
Care Management	Enhanced management and reduced mortality.	Parental anxiety regarding home monitoring. Sustainability and parental concerns with wearable biosensors.	Provide parental support and education for home monitoring with specialized nurses. Develop intelligent textile medical devices for comfort and continuous monitoring with robust data on security.
	Home monitoring programs for interstage care.		
	Use of wearable biosensors for continuous monitoring.		
Prevention	Addressing neurodevelopmental deficits.	Lack of data on infants and toddlers.	Conduct research on wearable biosensors in infants and toddlers in neuroevaluation.
	Continuous monitoring for early neurological event detection.	Parental anxiety over monitoring.	Address parental concerns through education with specialized nurses and parental association.
	Telehealth for neurodevelopmental assessments.	Addressing the digital divide for equitable access.	Bridge the digital divide for underserved communities.

## Data Availability

Not applicable.
